# The need for open access and natural language processing

**DOI:** 10.1073/pnas.2200752119

**Published:** 2022-04-04

**Authors:** Louis M. Barbier, James L. Green, David S. Draper

**Affiliations:** ^a^Office of the Chief Scientist, NASA, Washington, DC 20546

In PNAS, Chu and Evans (1) argue that the rapidly rising number of publications in any given field actually hinders progress. The rationale is that, if too many papers are published, the really novel ideas have trouble finding traction, and more and more people tend to “go along with the majority.” Review papers are cited more and more instead of original research. We agree with Chu and Evans: Scientists simply cannot keep up. This is why we argue that we must bring the powers of artificial intelligence/machine learning (AI/ML) and open access to the forefront. AI/ML is a powerful tool and can be used to ingest and analyze large quantities of data in a short period of time. For example, some of us (2) have used AI/ML tools to ingest 500,000+ abstracts from online archives (relatively easy to do today) and categorize them for strategic planning purposes. This letter offers a short follow-on to Chu and Evans (hereafter CE) to point out a way to mitigate the problems they delineate.

AI/ML has become a powerful tool in science and engineering, used in a multitude of applications (for example, image analysis and voice recognition). In this letter, we are focusing on natural language processing (NLP). NLP, a branch of AI, is well described in many places [see, for example, Mishra and Kumar (3)]. Commercial or custom-built NLP tools provide an elegant way to ingest and analyze large amounts of text in an extremely short period of time. The beauty of such an approach is that NLP, properly trained, can extract key words, organize text into topics, group topics and papers together, and identify outliers with relative ease. The key to using NLP in this way, however, relies heavily on “open science”; the corpus of papers in any given field must be available in an organized online archive that is searchable.

Only some simple steps need to be followed: identify the appropriate archive of published papers, train the NLP code using a subset of the archived papers, build “white” and “black” lists to help narrow the focus, and use the NLP to categorize and group papers by similarity and topic “closeness.” Network diagrams can help find linkages easily between papers where it may not have been obvious and, more to the point of CE (1), can easily show the “outliers”—the papers which stand apart and which might otherwise be missed.

In conclusion, we agree with CE (1) on the problems caused by the rapid rise in scientific publications, outpacing any individual’s ability to keep up. We propose that open access, combined with NLP, can help effectively organize the literature, and we encourage publishers to make papers open access, archives to make papers easily findable, and researchers to employ their own NLP as an important tool in their arsenal.
